# Unexpected self-lofting and dynamical confinement of volcanic plumes: the Raikoke 2019 case

**DOI:** 10.1038/s41598-022-27021-0

**Published:** 2022-12-27

**Authors:** S. M. Khaykin, A. T. J. de Laat, S. Godin-Beekmann, A. Hauchecorne, M. Ratynski

**Affiliations:** 1 Laboratoire Atmosphères, Observations Spatiales (LATMOS), UVSQ, Sorbonne Université, CNRS, IPSL, Guyancourt, France; 2 Royal Netherlands Meteorological Institute, De Bilt, The Netherlands

**Keywords:** Atmospheric dynamics, Atmospheric chemistry

## Abstract

Recent research has provided evidence of the self-lofting capacity of smoke aerosols in the stratosphere and their self-confinement by persistent anticyclones, which prolongs their atmospheric residence time and radiative effects. By contrast, the volcanic aerosols—composed mostly of non-absorptive sulphuric acid droplets—were never reported to be subject of dynamical confinement. Here we use high-resolution satellite observations to show that the eruption of Raikoke volcano in June 2019 produced a long-lived stratospheric anticyclone containing 24% of the total erupted mass of sulphur dioxide. The anticyclone persisted for more than 3 months, circumnavigated the globe three times, and ascended diabatically to 27 km altitude through radiative heating of volcanic ash contained by the plume. The mechanism of dynamical confinement has important implications for the planetary-scale transport of volcanic emissions, their stratospheric residence time, and atmospheric radiation balance. It also provides a challenge or “out of sample test” for weather and climate models that should be capable of reproducing similar structures.

## Introduction

Sulphur dioxide (SO_2_) from explosive volcanic eruptions is an important source of stratospheric aerosol via photochemical conversion of SO_2_ to sulfuric acid droplets (H_2_SO_4_). Strong volcanic eruptions such as the major eruption of mount Pinatubo in 1991 can inject vast amounts of volcanic material—mostly ash and SO_2_—high into the stratosphere which temporarily cools the climate^1^. Since the major eruption of Pinatubo, the most sizable moderate eruptions^2–4^ in terms of their impact on stratospheric aerosol load are Sarychev 2009, Nabro 2011, Calbuco 2015, Raikoke 2019 and Hunga 2022.

The 22 June 2019 eruption of the Raikoke volcano in the central Kuriles between Japan and Kamchatka (48.3° N, 153.4° E) was among the strongest explosive eruptions in the last three decades^5,6^ with the emitted SO_2_ mass between 1.4 and 2.1 Tg^7–9^ together with 3–15 Tg of fine-mode ash^10^, of which 1.5 ± 0.2 Tg of SO_2_^11–13^ and 0.4–1.8 Tg of fine ash^14^ have been injected directly into the lower stratosphere, to altitudes between 14 and 17 km^11–13,15^. While the volcanic cloud has rapidly spread over the northern Pacific, several isolated SO_2_-filled structures became clearly discernible in high-resolution SO_2_ imaging data soon after the eruption^9,15^. One of these structures could be tracked in satellite data as a coherent SO_2_ and/or aerosol structure for months and showed a remarkable diabatic climb by around 10 km or 140 K potential temperature in 2–3 months time, whilst moving equatorward^15^. While this climb is consistent with the rising branch of the Brewer-Dobson circulation^16^, it appears to be much faster (approximately 1.3–1.9 mm s^−1^) than the typical vertical velocity of 0.2–0.4 mm s^−1^ within this rising branch^17,18^. Given these ascent rates it would take approximately one full year to reach the reported rise in potential temperature or physical height. This points to the presence of diabatic self-lofting through the radiative heating of absorbing aerosols, as reported for the wildfire smoke plumes in the stratosphere using observations and modeling^19–24^.

Several recent studies^22,24–26^ have presented evidence that the rising smoke plumes are subject to dynamical self-confinement by synoptic-scale anticyclonic vortices that form around the plume, preventing them from stretching and diluting, thereby maintaining the absorbing aerosols at high concentration, which in turn provides a high degree of internal heating. The unusual behavior of Raikoke plume, in particular its persistence as a coherent structure, raises a question of whether a similar mechanism could be at play for volcanic plumes, which have not been previously reported to be subject of dynamical confinement.

In this study we combine various types of high-resolution observations and ECMWF meteorological reanalysis to explain the unexpected behavior of Raikoke emissions into the stratosphere.

## Results

### Anticyclonic confinement of volcanic material

**Figure 1:**
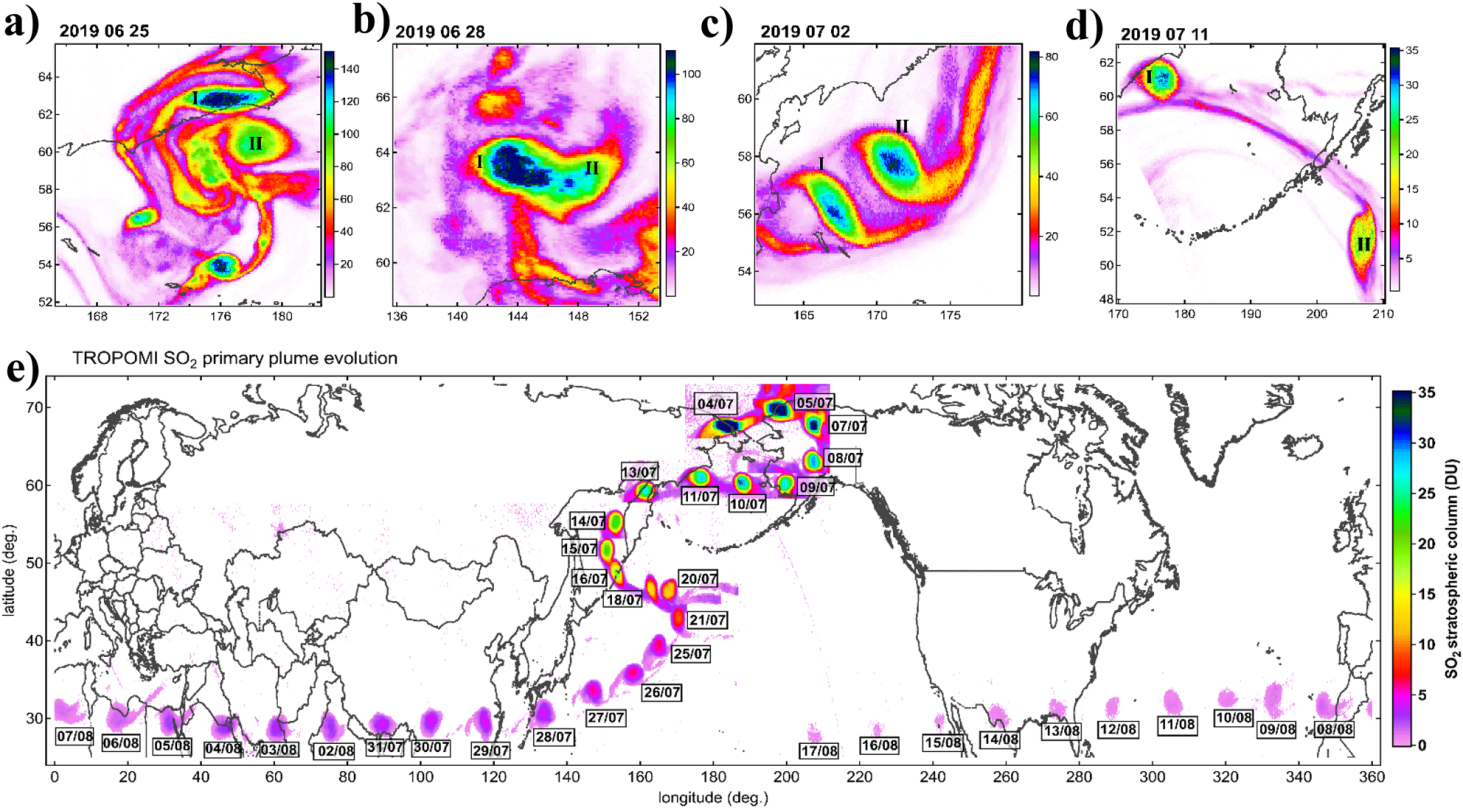
Evolution of the confined volcanic plume (structure I) from TROPOMI high-resolution mapping of SO2 stratospheric column in DU. (**a,b,c,d**) Early evolution of the Raikoke stratospheric SO plumes between 26 June and 11 July 2019. The two largest circular structures are denoted I and II. Note the different color scale of the panels. (**e**) Spatiotemporal evolution of the primary vortex (I). The dates in 2019 are denoted in dd/mm format.

Already on 25 June, three days after the eruption, the SO_2_ plume started to cluster into distinct isolated structures clearly discernible in high-resolution TROPOspheric Monitoring Instrument (TROPOMI) SO2 maps (Fig. 1a, see also Fig. S1 for the complete SO_2_ map sequence). The two largest structures, denoted I and II, appear to merge on 28 June (Fig. 1b) then separate again on 1 July (Fig. 1c) but continue to move alongside each other during the first week of July (Fig. 1c,d). The structure II eventually moved towards the Canadian Arctic^9^, whereas the structure I moved towards the Asian continent. Here we focus on the evolution of the structure I that showed remarkable stability and could be followed by various satellite sensors for months ahead^3,9,15^.

Figure 1e shows the spatiotemporal evolution of the structure I starting on 4 July, after it had traveled over northeast Siberia (Fig. S1). The plume then made a u-turn over Alaska, crossed the Northern Pacific and returned close 54%to its source location by mid-July. During the second half of July, the SO_2_ plume is observed as an isolated inverted comma-shaped structure with a circular core moving along the Eastern flank of the Asian Summer Monsoon Anticyclone before entering the subtropical jet, which rapidly advected the structure all across Eurasia and North Africa in 10 days. Over time, the maximum column amount of SO_2_ in the plume gradually decreases by photochemical processes that convert SO_2_ into H_2_SO_4_ droplets with an e-folding timescale between 8–18 days^15^ and 13–17 days^9^. Nevertheless, having preserved its compact shape, the plume could be followed in high-resolution TROPOMI SO_2_ total column measurements for 8 weeks.

**Figure 2:**
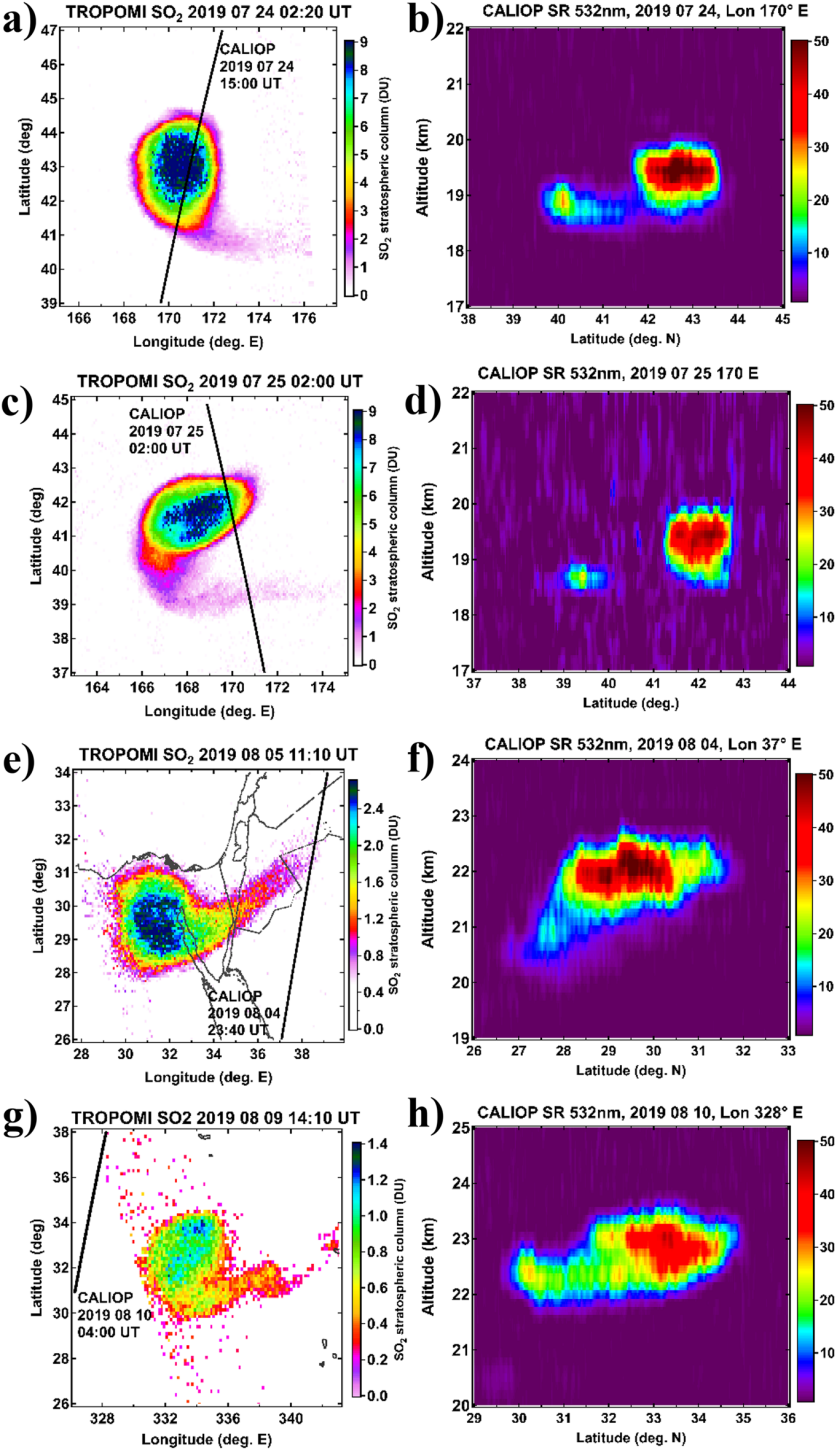
Horizontal and vertical structure of the primary VVP (vorticized volcanic plume). Left column (panels **a,c,e,g**): selected TROPOMI SO_2_ maps revealing the comma-shaped rotating plume. The nearest CALIOP orbit with indication of date/time is shown as black curve. Right column (panels **b,d,f,h**): corresponding CALIOP scattering ratio cross-sections through the plume. Panels **a,f,h** show CALIOP nighttime measurements that are more than 10 h apart from TROPOMI sampling. Panel **d** shows CALIOP daytime measurement precisely matching TROPOMI in time.

During its entire lifetime, the primary SO_2_ plume (structure I) maintained a circular shape, with clear signs of continuous filamentation (erosion) at the edges as shown in Fig. 1e, and Fig. 2a,b,c. A similar behaviour could be observed for the structure II [Ref.^9^, their Fig. 15b]. The compact circular shape is indicative of dynamical confinement, whereas the counterclockwise filamentation (or tailing) reveals the clockwise (anticyclonic) rotation of the plume. The filamentation at the edge of vortical structures is a common phenomenon in fluid dynamics associated with a vortex, especially in stably-stratified rotating fluids^27,28^. Indeed, it appears physically implausible that the compact shape of the plume could be preserved over such a long time in a highly dispersive environment^9^ without a dynamical confinement mechanism, already demonstrated for the stratospheric smoke plumes^22,24–26^. The anticyclonic motion of the two largest volcanic plumes is further corroborated by Aeolus satellite wind measurement and ECMWF ERA5 reanalysis. The phenomenon is hereinafter referred to as vorticized volcanic plume, VVP.

### Vertical structure and composition of the VVP

The high-resolution transects through the primary VVP from late July to mid-August (Fig. 2, right column) by the Cloud-Aerosol Lidar with Orthogonal Polarization (CALIOP) reveal a compact cloud of aerosol retaining a fairly compact shape over time and showing clear indication of the bottom-side elongation (tailing), characteristic of rotational motion of a plume^22^. The tail is clearly visible in the corresponding TROPOMI horizontal sections of the VVP (Fig. 2, left column) and although the CALIOP nighttime transects are more than ten hours out of phase with TROPOMI, complicating the cross-attribution of TROPOMI and CALIOP structures, the persistent anticyclonic filament observed by TROPOMI is most likely associated with the bottom-side elongation revealed by CALIOP. Indeed, the daytime CALIOP transect through the VVP (Fig. 2d)—coinciding precisely in time with the TROPOMI sampling (Fig. 2c)—reveals prominently the head and the tail of the structure.

According to CALIOP data, the primary VVP had a vertical extent of 1–1.5 km, whereas the horizontal extent of the associated SO_2_ structure increases from 200 to 300 km in early July to nearly 400 km by late July, that is when the VVP gets entrained by the subtropical jet. The horizontal extent of the VVP is thus two orders of magnitude larger than its vertical extent (aspect ratio or diameter over thickness of 133:1 to 400:1), or in other words, the VVP is a flat lens-like structure maintaining its shape for a long time. We note that the aspect ratio of the 6-wk old Raikoke VVP is about 3 times smaller compared to that of the largest smoke-charged vortices (SCV) produced by the Canadian wildfires in 2017^26^ and Australian bushfires in 2019/2020^22^.

Despite the very narrow vertical extent and the synoptic-scale horizontal extent of a few hundred kilometers, the primary VVP contained as much as 0.3 ± 0.1 Tg or 24% of the total SO_2_ mass injected into the stratosphere, as derived from TROPOMI data (Fig. S2). Similar estimate of SO_2_ mass is obtained for the secondary VVP (0.39 ± 0.12 Tg). Thus, around 54% of the Raikoke emission was initially contained by the long-lived vortices. While the shedding of volcanic material (presumably via the bottom-side filamentation) could have taken place, we note that the SO2 decay rate within the VVP structures did not change during the 8 weeks following the eruption, which suggests that the shedding was strongly limited by the dynamical confinement of the plumes.

### Aeolus observations of the VVP

**Figure 3:**
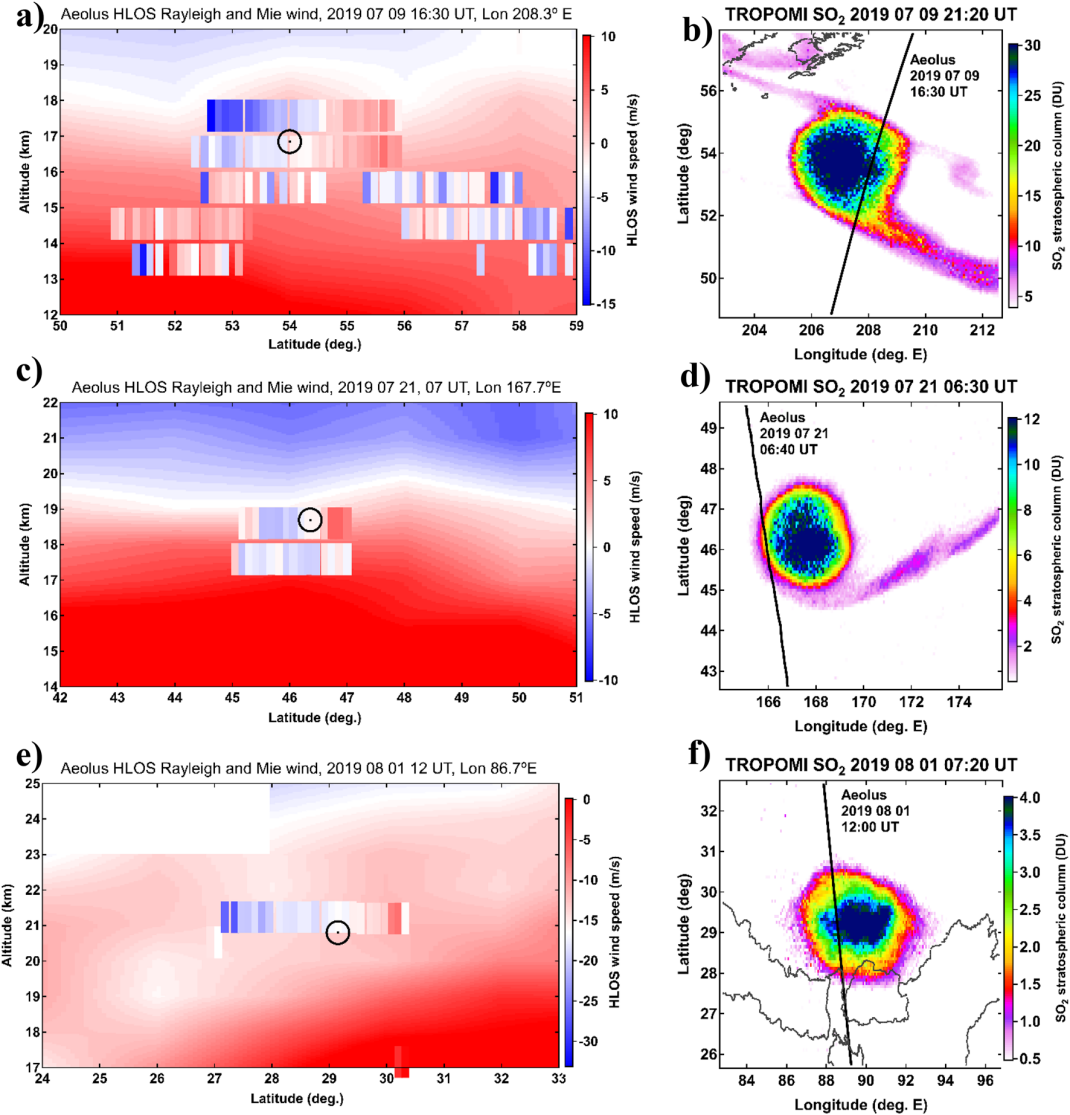
Observations of the secondary (top row) and primary (middle and bottom rows) VVP structures by Aeolus satellite Doppler wind lidar. (left column, **a,c,e**) Selected latitude-altitude sections of HLOS (quasi-zonal) wind velocity measured inside the rotating aerosol plume using Mie channel (vertically-elongated pixels) and background wind flow from Rayleigh channel (underlay) obtained by averaging all Aeolus Rayleigh profiles within a 3-d/30° longitude window (around 16 profiles per 1 degree latitude). The black circle marks the latitude-altitude location of the aerosol plume from CALIOP/OMPS-LP tracking. (Right column, **b,d,f**) corresponding maps of the confined SO2 plume from TROPOMI measurements and Aeolus orbit (black curve) with indication of date/time. The white blanked out area in panel e is due to ALADIN tropical range bin setting (see “Methods”).

While the persistent compact shape and circular filamentation of the SO_2_ structures are indicative of the vortical motion, direct observational evidence for their rotation is exclusively provided by ALADIN wind lidar onboard Aeolus satellite^29,30^ that sampled both VVPs several times during its lifetime. Figure 3 displays three cases of Aeolus sampling across the 17-d, 29-d and 40-d old VVPs. Aeolus measures the horizontal wind component transverse to the orbital plane, i.e. a quasi-zonal component except at high latitudes, using both molecular (Rayleigh) and particulate (Mie) backscattering (see “Methods”). The left-hand panels in Fig. 3 show the vertical cross sections of the Mie wind measured inside the VVP structures superimposed on the background flow derived by aggregating all Rayleigh winds within a 3-d/30° longitude window. The right-hand panels in Fig. 3 display the corresponding TROPOMI SO_2_ structures.

The compact Mie-wind feature between 16 and 18 km surmounting the meridionally more dispersed aerosol plume in Fig. 3a reveals a very particular horizontal gradient of wind speed and direction, changing from easterly at the southern edge to westerly at the northern edge, in a notable contrast with the near-zero background flow, thereby providing a clear indication of the anticyclonic motion of the plume. Note that the underlying, non-compact aerosol plume, resolved by ALADIN Mie channel, does not reveal such anticyclonic pattern, and appears to either be advected with the background flow or reflect the local mesoscale flow.

A similar Mie-wind feature, albeit of smaller horizontal extent, is shown in Fig. 3c. The limited horizontal extent is due to the fact that ALADIN sampled the edge of the vorticized plume, as shown in Fig. 3d. Another case presented in Fig. 3e reveals a prominent anticyclonic pattern of the primary vortex already at 21–22 km altitude (VVP self-lofting is discussed hereinafter). In all the three cases (and in the other ones not shown) the absolute wind speed increases towards the structure’s edges, which is exactly what one would expect for a rotating vortex. The anomalous wind speed at the edges (i.e. with respect to the background flow) is estimated at 9 ± 4 m s^−1^, which translates into 38 h revolution time, surprisingly similar to that of the largest Australian SCV with the 36-h revolution time^22^.

Additional evidence for the vortex was provided by an upper-air radiosounding at Hilo, Hawaii on 25 September, that is when a nearby lidar at Mauna Loa detected a strongly-scattering aerosol layer at 26 km altitude^3^ coinciding with a notable anomaly in zonal wind velocity reaching 15 m s^−1^ (Fig. S3).

### Diabatic lofting and circumglobal transport of the VVP

The three-dimensional tracking of the rising plume from mid-July to late-September 2019 was provided by Ref.^3,15^. Here we revisit this aspect and provide the VVP tracking since the eruption date (22 June) until mid-October from three satellite data sets: TROPOMI SO_2_ measurements of the circular plume during the first 8 weeks; CALIOP nighttime detections of the high scattering ratio anomaly as well as the Ozone Mapping and Profiler Suite (OMPS) Limb Profiler (LP) aerosol extinction profiles, enabling a robust tracking of the primary plume up to 15 weeks past the eruption.

**Figure 4:**
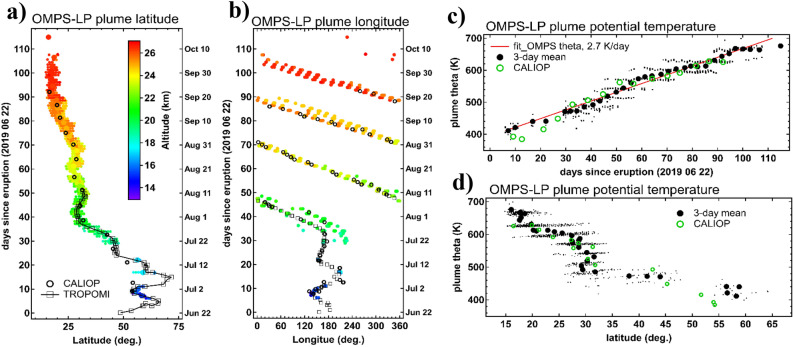
Circumglobal transport and diabatic lofting of the primary VVP. (**a,b**) Hovmoller diagrams of the VVP spatiotemporal evolution in zonal (**a**) and meridional (**b**) dimensions from TROPOMI (squares), CALIOP weekly averages (open circles) and OMPS-LP (filled circles with altitude color-coding). (**c,d**) Potential temperature of the VVP as a function of time (**c**) and latitude (**d**) from OMPS-LP individual detections (dots) and 3-d averages (black circles). CALIOP-derived weekly averages are shown as green open circles. Linear fit to OMPS-LP data is shown as red line in (**c**).

During the first three weeks of its lifetime, the primary VVP has changed the heading direction three times in the meridional plane (Fig. 4a) and seven times in the zonal plane (Fig. 4b) before entering the steady flow within the subtropical jet, which was followed by a triple circumnavigation and a progressive equatorward shift of the plume. The plume could be reliably tracked by satellite sensors for more than 100 days, during which it has traveled the distance of 139,000 km, that is the longest travel distance for a coherent aerosol plume (*cf.* 66,000 for the largest Australian SCV^22^).

The VVP tracking in the vertical dimension using aerosol vertical profiling by OMPS-LP and CALIOP are in good agreement, enabling an accurate estimation of the diabatic lifting rate of the stratospheric aerosol cloud. Figure 4c shows a steady diabatic ascent from around 400 K (15 km) to nearly 700 K (27 km) in three months with an average climb rate of 2.7 K day^-1^ (110 m day^−1^). This is notably lower than the mean diabatic rise rates for the Canadian SCV (5.6 K day^−1^)^20^ and Australian SCV (5.9 K day^−1^) ^22^. The ascent of the Raikoke VVP, although mostly linear in time, showed variations in its climb rate between 1.5 and 4.5 K day^−1^ (Fig. S4). Figure 4d suggests that the diabatic ascent in the latitude-altitude plane occurred in a stepwise manner, where the vertical steps correspond to the overpasses above the Asian monsoon region (Fig. S4). It is conceivable that the abundant highly-reflective convective clouds in the Asian monsoon region^31^ provided additional heating to the plume thereby accelerating its diabatic rise. Indeed, the multiple scattering of shortwave radiation over optically-thick convective clouds can result in significant warming of aerosols plumes^32–34^.

### Internal heating of the VVP

**Figure 5:**
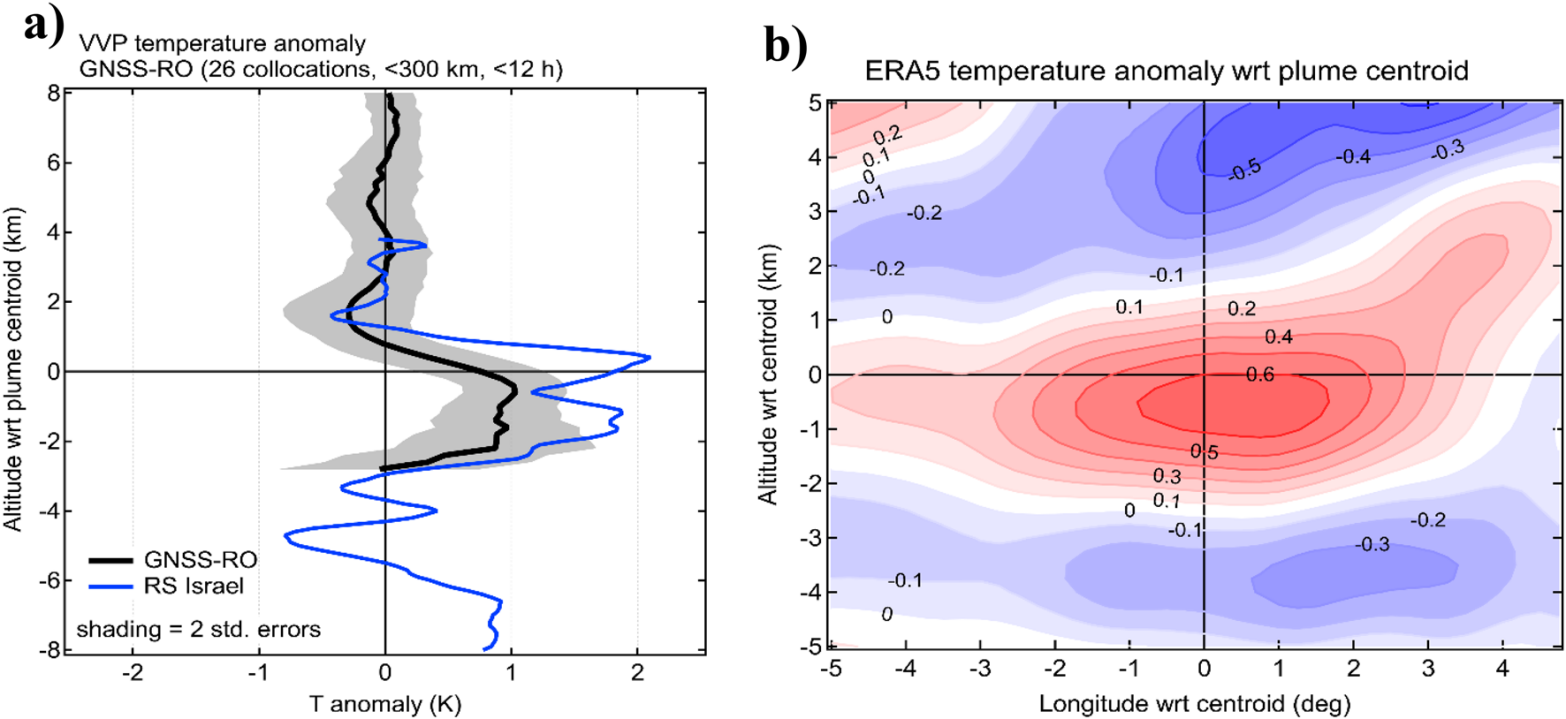
Internal heating of the VVP. (**a**) Composited temperature anomaly profile with respect to the aerosol plume centroid from 26 GNSS-RO measurement collocated with the plume during 25 July–10 August period. Grey shading represents two standard deviations. Blue curve shows the meteorological radiosounding from Bet Dagan station (Israel) that sampled the VVP on 5 August at 10 UTC (see Fig. S5). The temperature anomaly was computed with respect to 5-day average temperature profile. (**b**) Composited temperature anomaly in K from ERA5 reanalysis in the longitude-altitude plane with respect to the satellite-derived aerosol plume centroid. The composite is based on the data between 25 July and 10 August, that is when the vortex for overpassing above Asia and Africa for the first time.

In order to quantify the magnitude of internal heating, we used global navigation satellite system (GNSS) radio occultation (RO) temperature profiles collocated in space and time with VVP detections by OMPS-LP and CALIOP during the mid-July–early-August period when the VVP was transiting across Asia. Figure 5a displays a composited profile of temperature anomaly in the vertical coordinates relative to the plume centroid computed as deviation from the background temperature profile. The GNSS-RO measurements reveal a statistically-significant 2-km thick warm anomaly reaching 1 K near the plume’s centroid. Additional evidence for the aerosol plume heating was provided by an upper-air sounding in Israel on 5 August that sampled the VVP and revealed a 3 K warm anomaly (blue curve in Fig. 5a). The warm anomaly matches well with the stratospheric aerosol cloud detected by a nearby MicroPulse Lidar NETwork (MPLnet) lidar at Sede Boker, Israel (Fig. S5).

The horizontal extent of the warm anomaly was derived from ERA5 reanalysis which assimilates the GNSS-RO measurements. The longitude-altitude section of the temperature anomaly in ERA5 (Fig. 5b) agrees with the observed structure of the temperature anomaly and suggests its horizontal extent of 510 km, which is consistent with the diameter of the SO_2_ circular cloud estimated at 400 km.

The ERA5-derived warm anomaly reaching + 0.7 K is readily comparable with the heating of + 0.5 K reported for the Canadian SCV^26^ yet considerably smaller than + 4 K associated with the Australian SCV^22^. The heating of smoke plumes is mainly caused by the highly-absorptive black carbon particles. The volcanic plumes are primarily composed of weakly-absorbing sulfuric acid droplets produced by SO_2_ oxidation as well as the stronger-absorbing ash particles^2^. Estimates of the fraction of aerosol backscatter by ash particles inside the VVP, derived from CALIOP backscatter and depolarization (following Ref.^35^) suggest a high ash backscatter fraction in the young VVP of up to 40% gradually decreasing to zero on a timescale of one month (Fig. S6), most likely due to progressive sulfate coating of ash particles^35,36^. A similar decay was observed for the other, non-rising plumes, however their ash backscatter ash fraction was a factor of two smaller compared to the rising VVP (Fig. S6). The evolution of the Angstrom exponent derived from the OMPS-LP multiwavelength extinction measurements^37^ corroborate the reduction in the ash fraction.

While the diabatic self-lofting of the Raikoke confined plume was reported by Ref.^3,15^, here we provide the first observational evidence of the internal heating. The amount of internal heating caused by absorbing aerosols largely determines the diabatic lofting rate, however to maintain the heating at high degree for an extended time period, the absorbing material in the plume must remain at high concentration. This is conditioned by the dynamical confinement, which is a prerequisite for the VVP occurrence.

### Potential vorticity of the VVP

The occurrence of long-lived anticyclonic structure such as the VVP suggests that some form of fluid dynamical conservation mechanism is at work here, of which potential vorticity (PV) is a likely candidate given the rotational nature of the VVP. The PV is to first order a conserved atmospheric quantity in the absence of diabatic processes. The VVP resides in the extremely stable summer stratosphere where the air masses with different PV tend not to mix. This way, an eruption-driven injection of large amounts of tropospheric air characterised by low PV directly into the high-PV stratospheric environment could trigger anticyclonic rotation as reported for the wildfire-generated SCVs that appear as low absolute PV kernels in the meteorological (re)analyses^25,26^.

Note that the successful replication of such structures by operational meteorological models (that do not account for the volcanic or wildfire-induced vertical transport into the stratosphere), is linked with the assimilation of temperature and/or wind profiling from satellites and weather balloons^22^.

**Figure 6:**
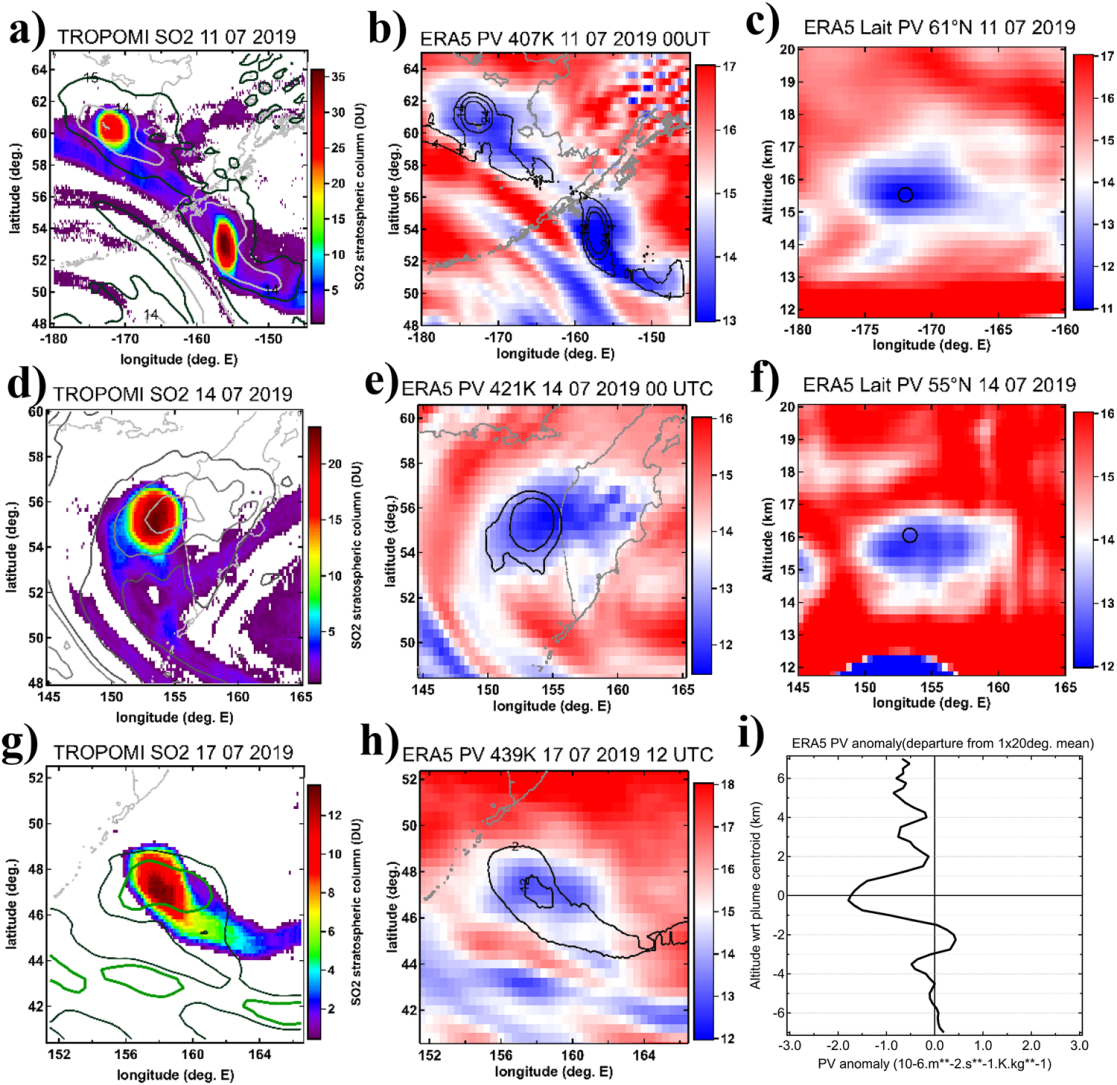
Potential vorticity of the vortex. (Left panels—**a,d,g**) TROPOMI maps revealing isolated compact SO2 structure associated with the vortex on three different dates in July 2019. Contours indicate the corresponding low potential vorticity anomaly. (Middle panels—**b,e,h**) ERA5 potential vorticity (PV in PVU, 1 PVU = 10^−6^ Km^2^ kg^−1^ s^−1^) interpolated to the potential temperature level of the aerosol plume centroid derived from satellite observations. Black contours mark the corresponding SO2 plume displayed in the left panels. (Right panels—**c,f**) Longitude-altitude sections of ERA5 Lait PV at the latitude of the primary VVP. The black circle marks the corresponding aerosol plume location derived from satellite observations. (**i**) Composited PV anomaly altitude profile with respect to the aerosol plume centroid obtained from ERA5 data between 25 July and 10 August. The PV anomaly is obtained as a deviation from an average PV profile within 1° latitude × 20° longitude bin.

In the case of Raikoke VVP, measuring 400 × 1.5 km at its maximum (i.e., 2–4 times smaller than the known SCVs^26^), the replication of such a small dynamical structure by meteorological analysis is more complicated due to limitations of the assimilated satellite observations. Nevertheless, the analysis of ERA5 data enabled identification of a low-PV anomaly associated with the VVP. Figure 6 reveals low-PV kernels (middle column) matching with the observed SO_2_ structures (left column). Similarly, the vertical PV cross sections (Fig. 6c,f) show a compact anomaly centered at the altitude of the satellite-derived aerosol plume. In Fig. 6a,b, one can see two distinct structures, of which the northern one is the primary VVP described in this study, whereas the southern one represents the secondary VVP (structure II in Fig. 1d) that was traveling side-by-side with the primary VVP during the first week of July before turning northeast. The low-PV structure associated with the primary VVP could be robustly followed in the reanalysis data until mid-July, becoming hardly discernible in ERA5 horizontal sections afterwards. Nonetheless, the composited vertical profile of PV anomaly derived by aggregating the reanalysis data from late-July to early August, when the VVP has been entrained by the subtropical jet, reveals a notable decrease of PV by 2 PVU symmetric to the aerosol plume centroid (Fig. 6i). A similar magnitude of PV anomaly (~ 3 PVU) was reported for the Canadian SCV^26^.

## Summary and discussion

In this study, we provided multiple lines of evidence that the compact circular shape of the rising plume was in fact maintained by a synoptic-scale anticyclone confining the cloud of SO_2_, ash and sulfate aerosols, that we termed the vorticized volcanic plume or VVP. The first line of evidence is provided by high-resolution SO_2_ measurements by TROPOMI, revealing a distinct circular shape—that could only be caused by rotative motion—together with filamentation at the edges (tailing) observed by TROPOMI and CALIOP, which is another attribute of rotation, demonstrated for the wildfire-generated vortices^22^. While the shape and the structure of the confined plume revealed by high-resolution observations are strongly suggestive of its anticyclonic motion, the direct and unequivocal observational evidence for the rotation was provided by Aeolus lidar wind profiling. Finally, the analysis of ERA5 data showed a localized low-PV anomaly associated with the plume, similar to what has been reported for the wildfire-generated SCVs^25,26^.

The Vorticized Volcanic Plume (VVP) produced by the Raikoke eruption persisted for more than three months, circumnavigated the globe thrice and lofted aerosols up to 27 km altitude, *i.e.* more than 10 km above the injection level. The plume contained about 0.3 Tg of SO_2_ (or 24% of the total SO_2_ eruptive mass), which was subject to self-lofting process—unusual for volcanic plumes—and remarkable longevity of more than three months. A more sizable midlatitude eruption like 1980 St Helens eruption in North America^38^ may be expected to generate bigger and more endurant dynamical structures, lending themselves for express lift of volcanic emissions through the stratosphere.

The longevity of the particular Raikoke plume addressed here has been first noted on the basis of CALIOP and ground-based lidar observations in Hawaii^3^. A more comprehensive tracking of the plume using various satellite instruments, including TROPOMI, pointed out its persistent compact circular shape^15^. Reference^15^ named it a coherent circular cloud (CCC) and two hypotheses regarding the confinement mechanism were proposed: a whirlpool (vortex) and a “dead fish” hypothesis, in which the plume was simply advected around by the background flow without being sheared apart because of the fairly quiescent environment of the summer stratosphere. That study^15^ did not find any clear indication for a vortex in the reanalysis wind fields and therefore preferred the dead fish hypothesis, in which the dynamical confinement is unnecessary to preserve the compact shape of the structure. However, another study^9^ did find strong evidence against the “dead fish” hypothesis by virtue of very dispersive lagrangian particle dispersion modelling results. Additionally, some indications for the anticyclonic circulation associated with the Raikoke rising plume were gleaned from geostationary visible-image animations and high-resolution radiosounding data^39^.

Another reason to assume that the VVP is a self-contained entity is the fact that the vortices were visible in TROPOMI data already on the third day after the eruption, when the volcanic material was still located at mid-latitudes. The strong stretching and dispersion of the volcanic material during the first few weeks after the event indicates that it was located in a dynamic and dispersive environment with high wind speeds^40^ that would be “hostile” to any non self-containing structure. Yet both structures apparently survive this environment while most of the other volcanic material rapidly disperses and spreads over thousands of kilometers within even days after the eruption^7,10^.

Dynamical confinement of stratospheric aerosol plumes by long-lived synoptic-scale anticyclones is a recently discovered phenomenon and an important advance in understanding of the aerosol plumes’ behavior in the stratosphere. It is also an interesting phenomenon for geophysical fluid dynamics, providing a new challenge for numerical modeling. The combination and coupling of the anticyclonic confinement and internal heating act to maintain the absorbing aerosols at high concentration, which lifts the cloud through radiative heating. As demonstrated above, this mechanism—already identified for the stratospheric smoke plumes—may as well apply to the volcanic plumes. We note that although satellite observations of stratospheric volcanic SO_2_/ash plumes and smoke from wildfires have been available for two decades, their use in studying and modelling the stratospheric dynamics has been limited if not mostly absent. Those observations thus could provide an untapped source of useful information for future research.

It is crucial to note that stratospheric injection of volcanic material does not necessarily lead to formation of stable anticyclones. As the PV gradient across the tropopause and the Coriolis factor, both increasing towards the poles (Fig. S7), are likely the key factors of the VVP formation, the tropical volcanic eruptions are much less likely to produce a self-confined anticyclone^41^. Moreover, since the internal heating of the plume aids in stabilizing the anticyclone, the absorbing properties of the emitted aerosols is another key factor. While the wildfire emissions include high-absorptive black carbon, the primary absorbing agent for the volcanic emissions is ash (tephra), hence the abundance of ash in the eruptive plume largely determines the lifetime of the VVP and its upward transport through the stratosphere. Finally, the background stratospheric conditions may also play a role since the persistence of a synoptic-scale anticyclone may be strongly limited by atmospheric wave activity, minimising during local summer. Otherwise stated, to generate a persistent anticyclone, a volcanic eruption must be rich in ash, occur in the extratropics and preferably during local summer. These environmental conditions are naturally satisfied for the wildfire-driven Pyrocumulonimbus (pyroCb) thunderstorms emitting absorptive carbonaceous aerosols however not necessarily for volcanic eruptions which occur randomly in space and time. The Raikoke eruption is nevertheless not unique in this respect and generation of dynamically-confined self-lofting plumes remains to be investigated for the other summer midlatitude eruptions such as the eruptions of Kasatochi in 2008 and Sarychev 2009. It is conceivable that similar structures have formed for the past volcanic eruptions but were more difficult to observe due to poorer satellite observational capacity. The disclosure of this phenomenon after the Raikoke event largely owes to the recent advances in the satellite-based observing systems and operational analysis.

It is worthwhile noting that the VVP has some similarities with the frozen-in anticyclones (FriACs) that occasionally occur in the Summer Arctic stratosphere^42,43^. However for the FriAC, the formation of the anticyclone is not due to the lifting of air by diabatic heating, but due to the entry of low-latitude air into the quiet high-latitude summer stratosphere at the time of the spring transition.

Finally, the findings presented have important implications for understanding the climate impact of explosive volcanic eruptions. Persistent anticyclonic formations maintain the volcanic plumes at high concentration thereby providing vertical thrust through internal heating of absorbing ash aerosols. The resulting diabatic lofting of the aerosol plumes not only prolongs their stratospheric residence time—mostly limited by slow gravitational settling of aerosol particles^2^—but also enhances the meridional dispersion of injected material^23^, thereby affecting the atmospheric radiative balance^44,45^ and ozone chemistry^46^. In addition, the mechanism of self-confinement and self-lofting of aerosol plumes should be accounted for in the geoengineering considerations related to solar radiation management.

## Methods

### TROPOMI

The TROPOspheric Monitoring Instrument (TROPOMI) is a nadir imaging instrument onboard ESA's Sentinel-5 Precursor satellite that was launched in October 2017. TROPOMI provides daytime measurements^47,48^ of various species as columnar densities with a swath width of 2600 km and a very high spatial resolution of 7 × 3.5 km^2^. Here we used the TROPOMI Level 2 offline (OFFL) V01.01.07 SO_2_ data product^49^ retrieved for the plume height above 15 km as it is considered to provide the best approximation for the Raikoke eruption as in the other studies^7,9^. The data were gridded to horizontal resolution of 0.075 × 0.075° after discarding the data points below the detection limit for TROPOMI (0.3 DU) and the data corresponding to solar zenith angle above 70°.

### CALIPSO CALIOP

The Cloud-Aerosol Lidar with Orthogonal Polarization (CALIOP) is a two-wavelength polarization lidar on board the Cloud-Aerosol Lidar and Infrared Pathfinder Satellite Observations (CALIPSO) satellite that performs global profiling of aerosols and clouds in the troposphere and lower stratosphere^50^. We use the total attenuated backscatter level 1B product V4.1. The along track horizontal/vertical resolution are respectively 1 km/60 m between 8.5 and 20.1 km, 1.667 km/180 m between 20.1 and 30.1 km. The data are resampled to a uniform horizontal/vertical resolution of 500 m/50 m. The scattering ratio (SR) is computed as the ratio between the total attenuated backscatter at 532 nm and molecular backscatter derived from the air density supplied with the L1B product. The particulate depolarization ratio (PDR) is computed from the total and perpendicular components of the attenuated backscatter. The plume detection and tracking is performed by locating the vertical layers of more than 750 m thickness with SR > 3 and PDR < 0.15 (to exclude high-level cirrus clouds). Only night-time CALIOP data are used for the plume detection because of the lower signal-to-noise ratio of the daytime acquisitions^50^. The fraction of backscatter due to ash particles is computed from SR and PDR^36^.

### OMPS-LP

The Ozone Mapping and Profiler Suite Limb Profiler (OMPS-LP) on the Suomi National Polar-orbiting Partnership (Suomi-NPP) satellite, which has been in operation since April 2012, measures vertical images of limb scattered sunlight in the 290–1000 nm spectral range^51^. The sensor employs three vertical slits separated horizontally to provide near-global coverage in 3–4 days and more than 7000 profiles per day with vertical resolution of 1—2 km in the stratosphere. Here we use OMPS-LP V2.0 aerosol extinction data^52^ at 675 nm for tracking the rising VVP. The tracking is performed by locating the stratospheric data with extinction ratio (computed as the ratio between aerosol and molecular extinction) ER > 8. Additional filtering of the resulting 3D track of the plume is done by comparing it with CALIOP and TROPOMI tracking.

### Aeolus ALADIN

The Atmospheric LAser Doppler INstrument (ALADIN) is a Doppler wind lidar operating at 355 nm onboard European Space Agency’s Aeolus mission launched in 2018 and providing near-global measurements of wind profiles from ground up to about 25 km^30^. ALADIN measures horizontal line-of-sight wind (HLOS) velocity (transverse to the orbital plane), that is a quasi-zonal component except at high latitudes. The HLOS wind is retrieved neglecting the vertical wind component by means of two different frequency discriminators, namely a double-edge Fabry–Perot interferometer sensing the Doppler-shifted broadband molecular (Rayleigh) backscatter signal and Fizeau interferometer analysing the narrowband particulate (Mie) backscatter signal. The Mie channel can only sense wind velocity in the presence of clouds and aerosols. The processing chain determines whether the scene is clear-sky or cloudy for each individual measurement and classifies the data into Rayleigh and Mie types by applying predefined thresholds on the signal-to-noise ratio. Both Rayleigh and Mie data are further classified into clear and cloudy types based on the scattering ratio (total-to-molecular backscatter coefficient ratio) of 1.4^53^. Here we use L2B (baseline L2B12) quality-screened Rayleigh-clear and Mie-cloudy data products. The vertical resolution of both Rayleigh and Mie channels varies between 0.25 and 2 km (~ 1 km in the lower stratosphere). The horizontal resolution is about 90 km (Rayleigh) and 10 km (Mie). ALADIN has dynamic range bin setting (RBS), which determines the maximum measurement altitude (and vertical resolution). The RBS is different for Rayleigh and Mie channels and varies with time and latitude. The latest estimates of ALADIN uncertainties using reference measurements reported systematic (random) error of − 0.8 m s^−1^ (5.4 m s^−1^) for the Rayleigh product^54^ and − 0.7 m s^−1^ (2.9 m s^−1^) for the Mie product^55^.

### GNSS-RO

We use Global Navigation Satellite System (GNSS) radio occultation (RO) dry temperature profiles acquired onboard Metop A/B/C satellites and processed at EUMETSAT RO Meteorology Satellite Application Facility (ROM SAF)^56^. The vertical resolution of RO temperature profiles is about 500 m in the lower stratosphere. For computing the composited temperature perturbation within the rising aerosol plume we use temperature profiles collocated with the plume centroid as identified using CALIOP/OMPS-LP tracking (12 h, 300 km collocation criteria). The perturbation is computed as the departure from a mean temperature profile (background profile) within the corresponding spatiotemporal bin (2-day, 2° latitude, 180° longitude).

### ECMWF ERA5

We use the European Center for Medium Range Forecasts ERA5 reanalysis^57^ at 0.4 × 0.4° horizontal resolution and L137 (model levels) vertical resolution with 12-hourly sampling. The Lait potential vorticity is calculated from Ertel potential vorticity (PV) as LPV = PV(Θ_0_/Θ)^9/2^, where Θ is potential temperature in K and Θ_0_ is the potential temperature of the plume centroid inferred from satellite plume detections. Note that ERA5 does not assimilate the aerosols in the stratosphere and therefore cannot account for their direct radiative effect.

## Supplementary Information

Supplementary Figures.

## Data Availability

The TROPOMI SO_2_ product data were obtained from the Copernicus Open Data Hub at https://s5phub.copernicus.eu/ (Last Access: 15 September 2022); CALIOP data are available at https://www-calipso.larc.nasa.gov/products/; OMPS data are available at https://snpp-omps.gesdisc.eosdis.nasa.gov/data/SNPP_OMPS_Level2/OMPS_NPP_LP_L2_AER_DAILY.2/2019/; GNSS-RO data at https://www.romsaf.org/product_archive.php; ALADIN data are available from ESA 230 at https://earth.esa.int/eogateway/missions/aeolus/data; ERA5 data are available at https: //www.ecmwf.int/en/forecasts/datasets/reanalysis-datasets/era5; radiosounding data are available at http://www.weather.uwyo.edu/upperair/bufrraob.shtml; MPLnet lidar data and quicklooks are available at https://mplnet.gsfc.nasa.gov/out/data/.
