# Supplementary material for: Unexpected self-lofting and dynamical confinement of volcanic plumes: the Raikoke 2019 case
Source: Sci Rep. 2022 Dec 27;12:22409. doi: 10.1038/s41598-022-27021-0 (PMC9794726; doi:10.1038/s41598-022-27021-0)
Supplement: Supplementary file 1 — Supplementary Figures. [file 41598_2022_27021_MOESM1_ESM.pdf]

Supplementary Material for

Unexpected self-lofting and dynamical confinement of volcanic plumes: the Raikoke 2019 case

S. M. Khaykin[1*], A.T. J. de Laat[2*], S. Godin-Beekmann[1], A. Hauchecorne[1], M. Ratynski[1]

[1] Laboratoire Atmosphères, Observations Spatiales (LATMOS), UVSQ, Sorbonne Université, CNRS, IPSL, Guyancourt, France

[2] Royal Netherlands Meteorological Institute, De Bilt, the Netherlands

Corresponding authors: Sergey Khaykin (sergey.khaykin@latmos.ipsl.fr), Jos de Laat (jos.de.laat@knmi.nl)

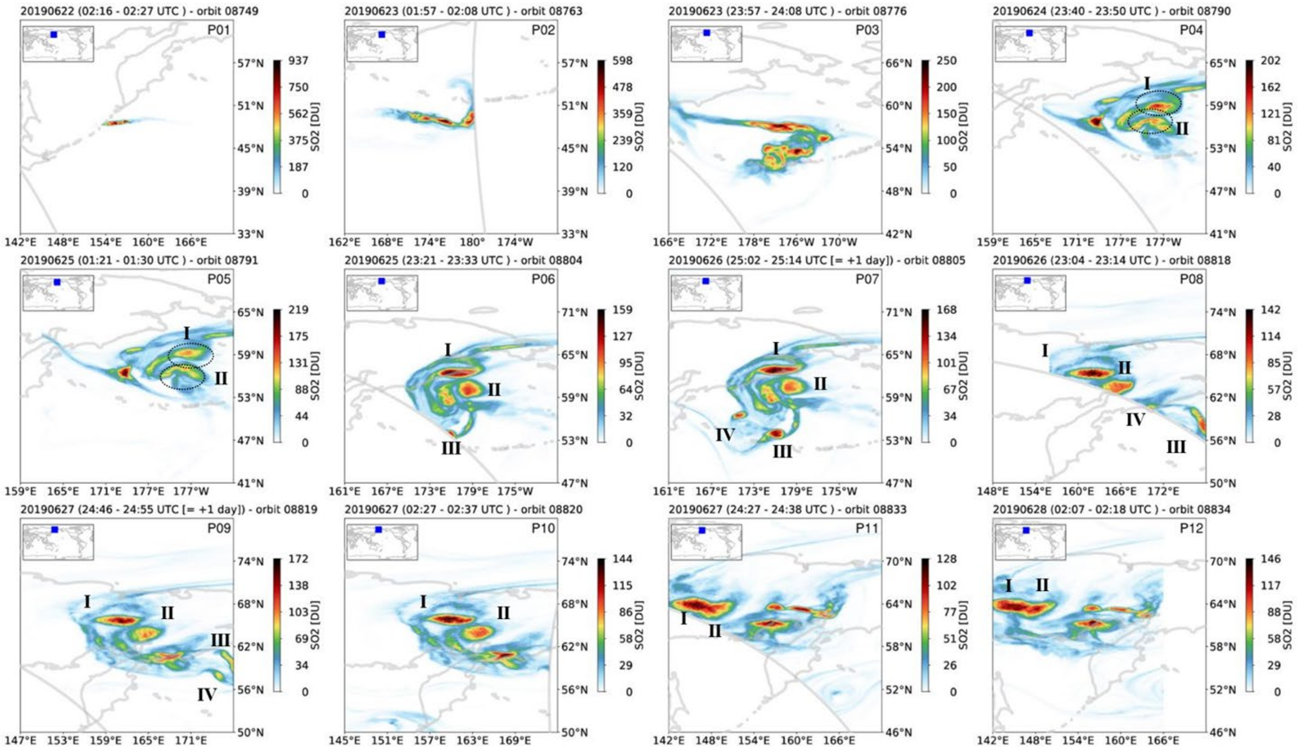

Figure S1a. Composite of twelve TROPOMI SO2 contour panels of the June 2019 Raikoke eruption plumes between 22 and 28 June 2019. Color intervals are dynamically determined based on the maximum SO2 column values within the scene and thus vary per panel. Two to four SO2-filled structures identifyiable in the imagery are labeled to allow for visual tracking. Note that because of the high latitude of the eruption plume, multiple overpasses by the polar orbiting TROPOMI satellite can occur.

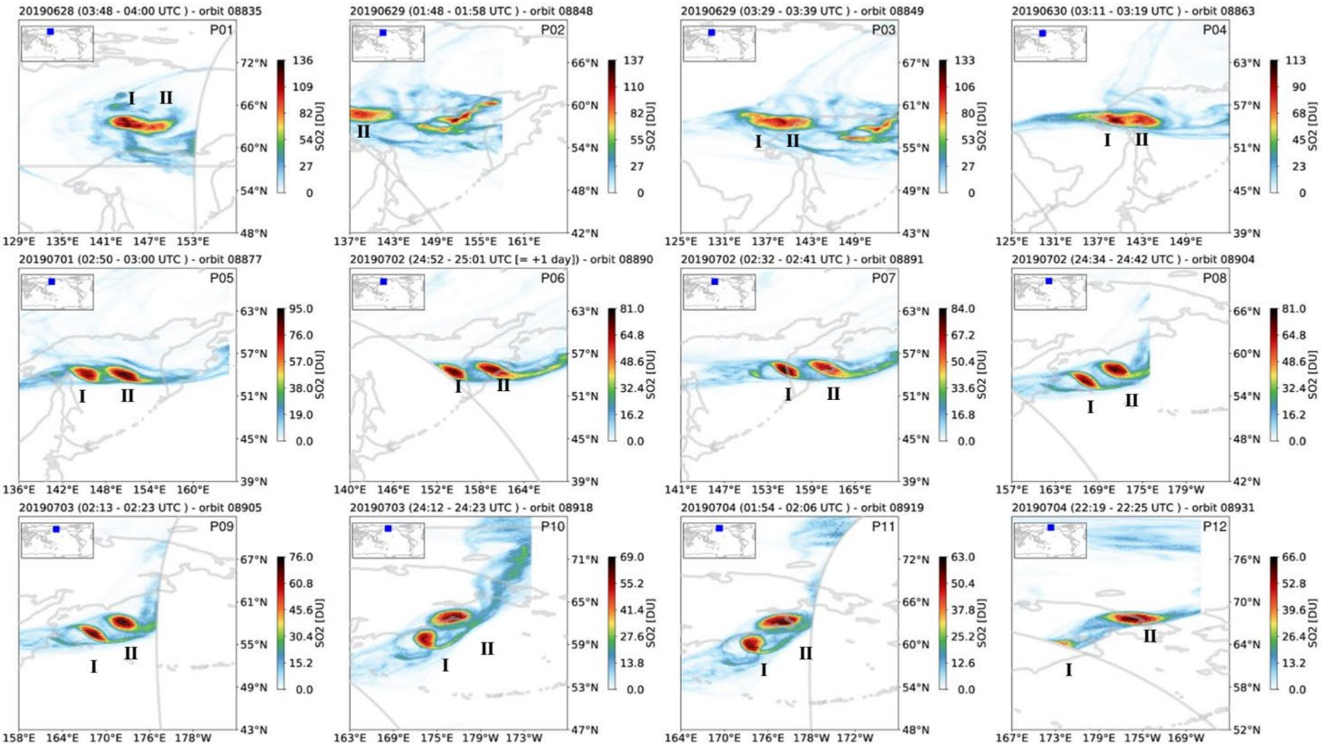

Figure S1b. As figure S1a but for scenes between 28 June 2019 and 4 July 2019

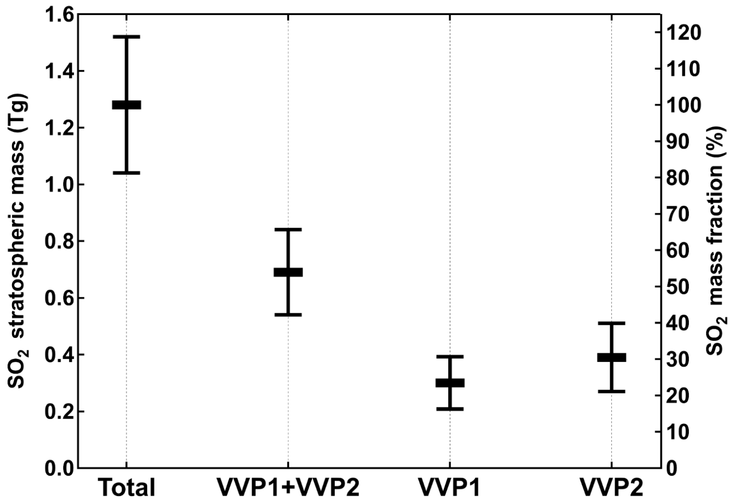

Figure S2. TROPOMI-based estimates of the SO_2 masses (left axis) injected by Raikoke eruption into the stratosphere for different parts of the plume: entire plume observed on 23-24 June 2019 (total) and isolated parts of the plume contained by the two vortices (VVP1 and VVP2 for the period 25 June - 25 July). As in Cai et al. (2020), the SO_2 masses are computed from daily TROPOMI data with account for the observed exponential SO2 decay due to its oxidation to H_2SO_4. The error bars represent two standard deviations. Right axis shows the percentage fraction of the various plumes with respect to the total mass of Raikoke-emitted SO_2.

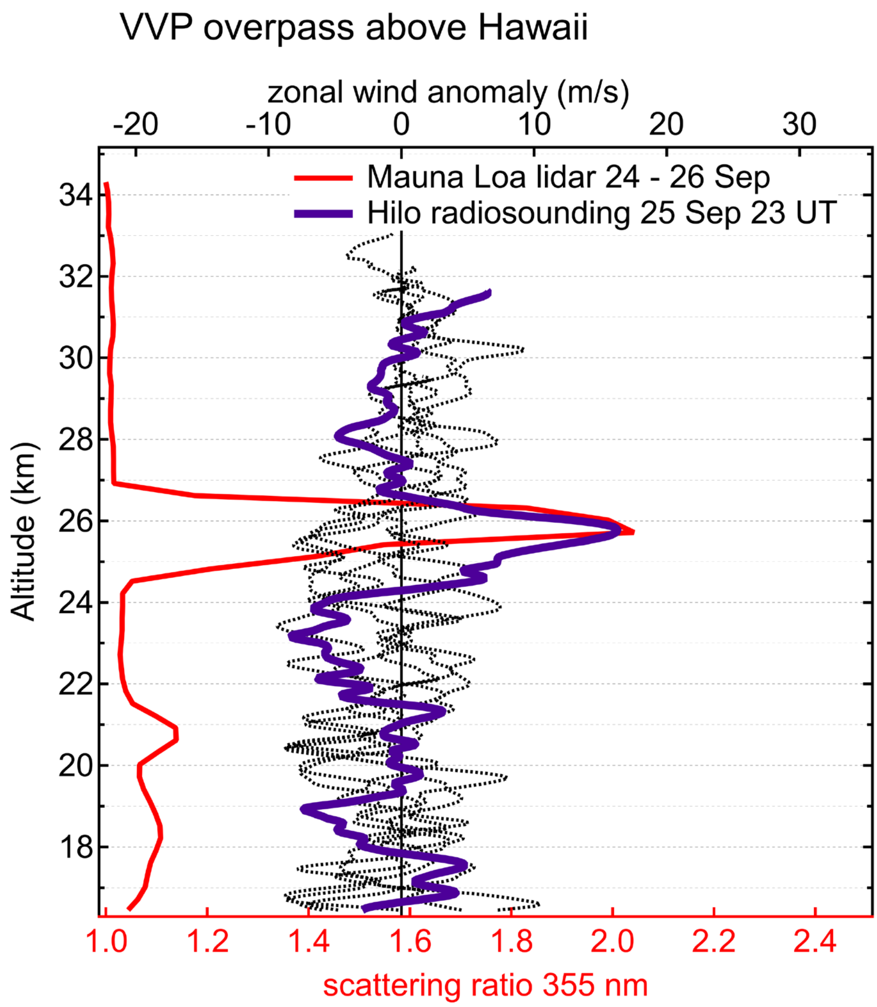

Figure S3. Observations of the vorticized volcanic plume above Hawaii by meteorological radiosounding at Hilo on 25 September 2019 (violet curve) revealing a zonal wind anomaly at 26 km altitude coinciding with an intense layer of aerosol (red curve) detected by Mauna Loa aerosol lidar (Chouza et al., ACP, 2020). The dashed black curves represent the zonal wind anomaly profiles from 23 to 27 September

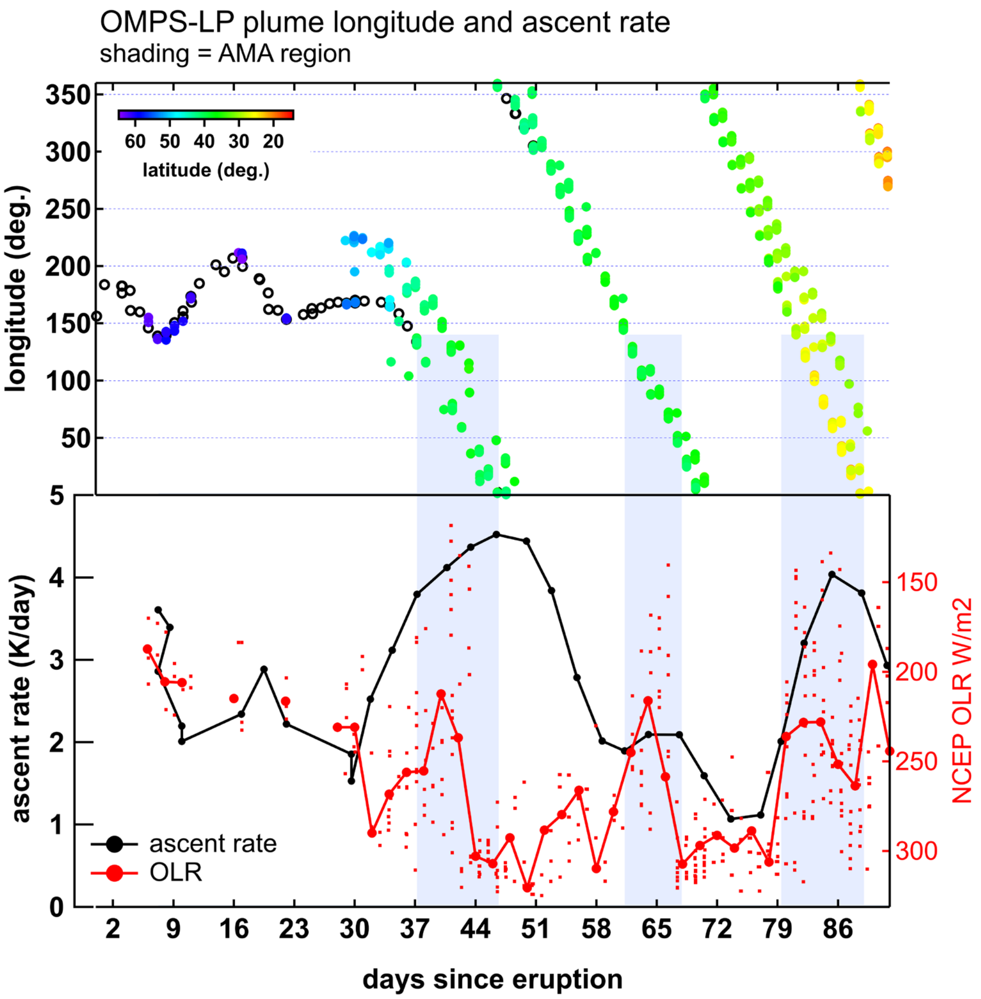

Figure S4. Evolution of the diabatic ascent rate of the vorticized plume from OMPS-LP aerosol extinction profiling. (top) VVP longitude evolution with color coding by the plume altitude. (bottom) VVP diabatic ascent rate evolution (3-d averages, black curve) and outgoing longwave radiation (OLR) from NCEP reanalysis data collocated with the VVP track (red dots - individual values, red curve - 3-d averages). Note that the OLR is inversely proportional to the deep convective activity, which is why the y axis is reversed. The shaded area indicate the periods when the VVP was passing above the Asian monsoon anticyclone region (defined as Montgomery stream function at 390-410 K as in Khaykin et al., ACP, 2022)

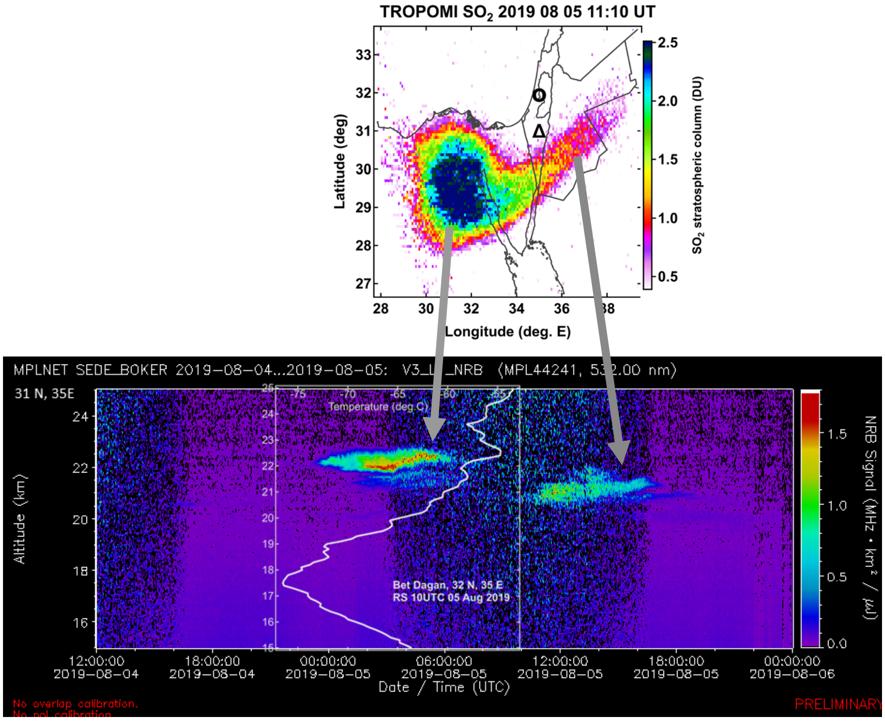

Figure S5. (top) TROPOMI map of the vorticized SO2 plume on 5 August during its overpass above Israelien Bet Dagan radiosounding station (marked as open circle on the map) and MPLnet/AERONET lidar site in Sede Boker (triangle). The circular core of the VVP and its filament (tail) are clearly visible. (bottom) Time-altitude curtain of normalized raw lidar signal from Sede Boker site showing the transit of the core of the plume at 22 km followed by the tail sampling at 21 km altitude. The lidar image is taken from MPLnet web site https://mplnet.gsfc.nasa.gov/data?s=SEDE_BOKER&v=V3

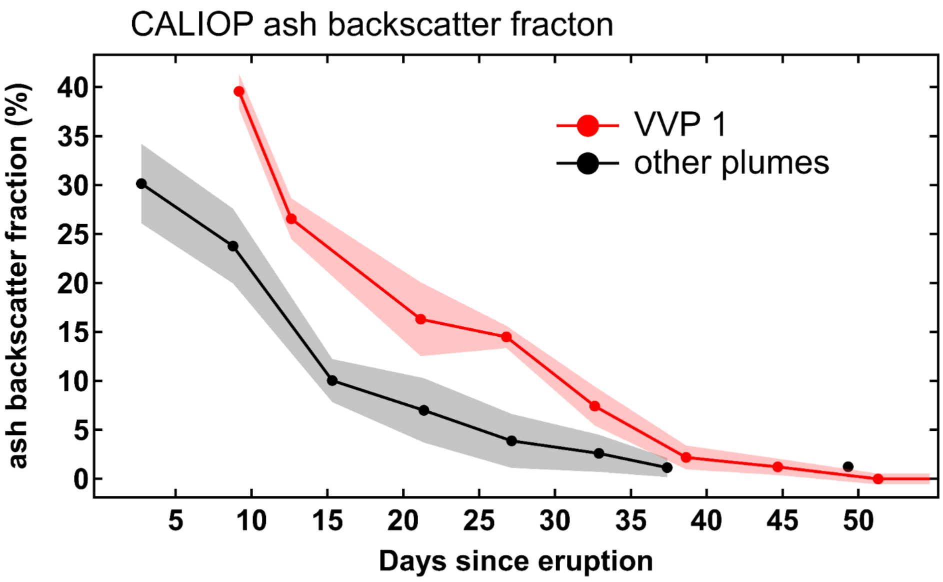

Figure S6. Fraction of backscatter due to ash particles in the Raikoke aerosol plumes (6-day averages) derived from CALIOP aerosol backscatter and depolarization measurements following the method described by Vernier et al. (2016) and references therein. The red curve corresponds to the primary vortex (VVP1), the black curve is based on the measurements within other plumes except the secondary vortex (VVP2). The shading represents two standard deviations of the 6-day averages.

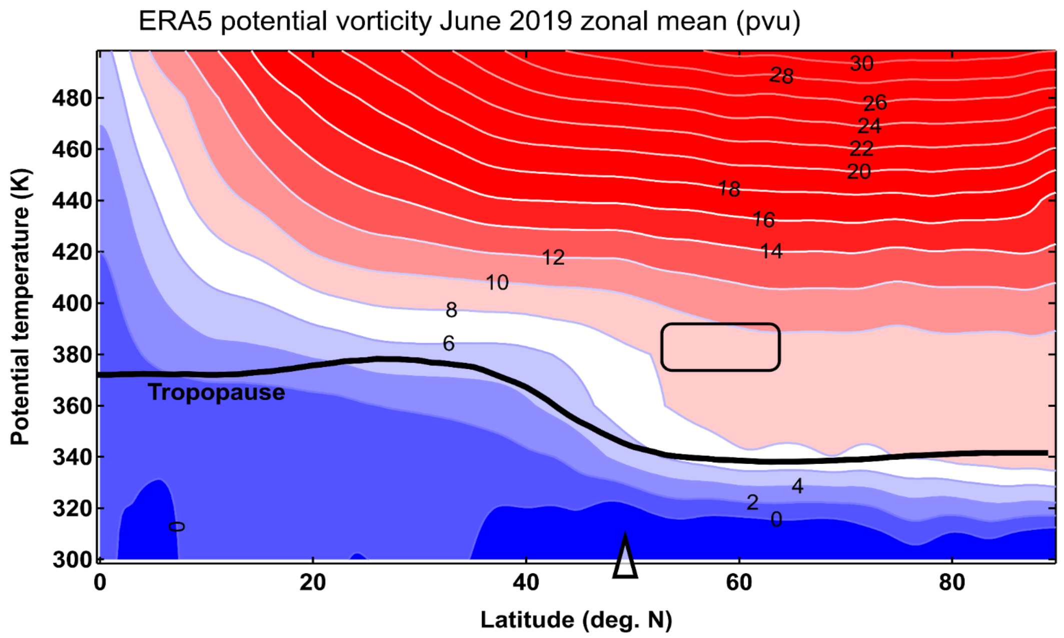

Figure S7. Latitude-altitude section of ERA5 potential vorticity in July 2019. The triangle on the bottom axis indicates the location of Raikoke volcano, the black rectangle marks the approximate location of volcanic plumes three days after the eruption. Note the poleward increase of the cross-tropopause PV gradient, suggesting that mid- and high-latitude injections of low-PV airmasses into the stratosphere result in a stronger difference in PV between the injected airmass and the higher-PV stratospheric environment.
